# Complex chronic respiratory disease concurrent with coccidiosis in broiler chickens in Malaysia: A case report

**DOI:** 10.5455/javar.2021.h547

**Published:** 2021-11-01

**Authors:** Intan Noor Aina Kamaruzaman, Kian Yiing Ng, Ruhil Hayati Hamdan, Nurshahirah Shaharulnizim, Che Wan Salma Che Wan Zalati, Maizan Mohamed, Muhammad Luqman Nordin, Nur Zul Izzati Mohd Rajdi, Luqman Abu-Bakar, Mohd Farhan Hanif Reduan

**Affiliations:** Faculty of Veterinary Medicine, Universiti Malaysia Kelantan, Kelantan, Malaysia

**Keywords:** Chronic, coccidiosis, diarrhea, *Escherichia coli*, respiratory

## Abstract

**Objective:**

The case study describes the cause of an increase in mortality rates among 35-day-old broilers that developed respiratory distress and bloody diarrhea on a farm in Malaysia.

**Materials and Methods:**

The organ samples were subjected to laboratory testing and postmortem inspection. *Escherichia *(E.)* coli* and *Mycoplasma *(M.) *gallisepticum* were detected using bacterial isolation and molecular diagnostics using polymerase chain reaction.

**Results:**

Chickens with the infection had widespread fibrin buildup in several organs and hemorrhages on the duodenal mucosa. Additional histology and laboratory analysis of organ samples revealed infection with *M. gallisepticum*, *E. coli*, and enteric *Eimeria* spp., all of which are consistent with complex chronic respiratory disease (CCRD) associated with coccidiosis. Tylosin tartrate 20% (w/w) (2.5 gm/l) was prescribed for 1 week along with a combination of the broad-spectrum bacteriostatic drug streptomycin (25 mg/kg) and coccidiostat (2 gm/5 l).

**Conclusion:**

CCRD and coccidiosis are both infectious diseases that can infect chicken flocks, resulting in production losses and carcass quality degradation. Early disease detection and proper treatment should be provided promptly, and tight farm biosecurity should be implemented to prevent chicken mortality on the farm, as was achieved successfully.

## Introduction

Chronic respiratory disease (CRD) is a frequent respiratory infection in chickens caused by *Mycoplasma* (*M.*)* gallisepticum* [1]. *M. gallisepticum*, which is found globally, is a significant mycoplasmal infection of avian species [2]. Recent investigations in Malaysia’s chicken farms revealed a significant frequency of *M. gallisepticum* infection [2,3]. Infection with opportunistic bacteria such as *Escherichia *(*E.*)* coli* complicates the organism’s pathogenicity, resulting in severe air sacculitis and septicemia, a condition known as complex chronic respiratory disease (CCRD) [1]. The CCRD has been shown to directly result in increased morbidity and mortality, as well as carcass condemnation and downgrading [4].

Coccidiosis is another significant illness in the chicken industry, caused by protozoan parasites of the genus *Eimeria*. This parasite infects and replicates within the intestinal epithelial cells of birds, resulting in decreased productivity and high treatment costs. Coccidiosis management costs the chicken sector an estimated £2 billion (USD 2.5 billion) in direct losses each year [5]. This article describes the co-occurrence of CCRD and coccidiosis in broiler chickens in Malaysia. Both illnesses are critical in broiler production, and as a result, these findings alarmed the farmer. Appropriate measures must be made to prevent pathogens from spreading to the flock.

## Materials and Methods

### Ethical approval

In this instance, no ethical approval is required. A postmortem was done on all dead chickens on the farm, with the farmer’s consent, as part of the disease investigation.

### Case detail

A broiler chicken farm in Malaysia with a total population of 63,000 birds (35-day-old) was diagnosed with respiratory problems and diarrhea, as well as a slightly higher mortality rate of >0.1% than usual. The birds were housed in an open house with a raised floor and had had multiple immunizations (infectious bronchitis, Newcastle disease, and infectious bursal disease) prior to the veterinary visit. When the birds were examined physically, they were dull, sluggish, and dejected. Uneven growth was found among flocks, and gasping and rales indicated respiratory discomfort. Additionally, most birds had pasty vents and watery feces (tinted with blood) on the house floor. A postmortem investigation of 16 deceased birds was performed.

## Results

### Gross pathology

**Figure 1.**
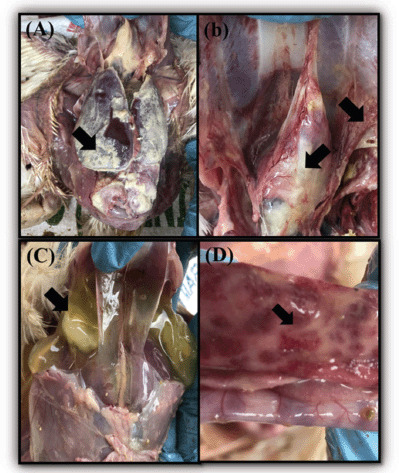
The postmortem findings of the necropsied chickens. (A) The generalized yellowish fibrinous coating on multiple organ surfaces of the heart, liver and peritoneal cavity indicating fibrinous inflammation. (B) Large amount of fibrinous materials deposited on the thoracic air sacs and pericardium layer of the heart with prominent and reddened blood capillaries indicating fibrinous and hemorrhagic inflammation (C) Yellowish fibrinous exudate with gel-like characteristic accumulated in the abdominal cavity of severely infected chicken (D) Thickened mucosa with the presence of multiple ecchymotic hemorrhages on the duodenum.

External examination of carcasses revealed emaciation and untidy feathers. Internal examination revealed a uniform yellowish coating across the surface of the heart and liver, consistent with fibrin deposition (Fig. 1A). Additionally, fibrous deposits were discovered on the bird’s air sac (Fig. 1B). Within the respiratory system, the trachea was constricted significantly. The heart seemed to be enlarged, and the pericardial sac had a fibrinous coating. Additionally, fluid containing clotted fibrin accumulated in the abdominal cavity (Fig. 1C). The gastrointestinal tract was examined grossly and revealed thickening and hemorrhages on the duodenal mucosa (Fig. 1D).

**Figure 2.**
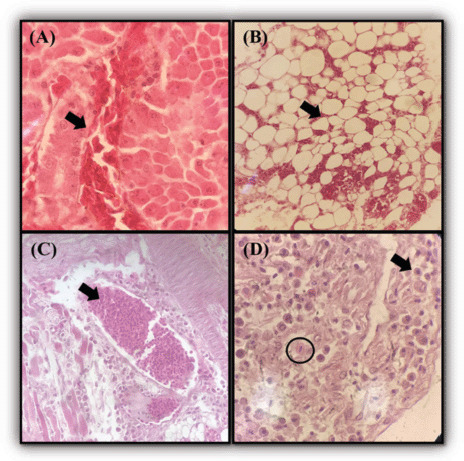
The histopathology findings of chicken organs. (A) vacuolization of the liver cytoplasm, loss of sinusoidal architecture suggestive for hepatic degeneration and necrosis. (B) The presence of distended alveolar space is suggestive of pulmonary emphysema. (C) Presence of inflammatory cells and blood cells on the vessel of the trachea indicating tracheitis and congestion (D) Presence of microgamete (marked by a black circle) and schizonts (marked by the red arrow) in the duodenum tissue suggestive for coccidiosis caused by *Eimeria *spp. Hematoxylin and Eosin staining 10× and 40×.

Numerous abnormalities were discovered during histopathological evaluation of the organ samples. The hepatic cells’ sinusoidal architecture was lost, implying liver degeneration and necrosis (Fig. 2A). Distension of alveolar gaps was observed in the lung tissue (Fig. 2B), indicating pulmonary emphysema. Tracheal tissue was densely packed with inflammatory and blood cells, consistent with tracheitis and congestion (Fig. 2C). The presence of schizonts and microgametes in the duodenum histologically suggested coccidiosis (Fig. 2D). Microbiological investigation indicated the presence of *E. coli* in all organ samples. Additionally, molecular testing of the liver, trachea, and lugs using an *M. gallisepticum* polymerase chain reaction (PCR) methodology reported by Mettifogo et al. [6] revealed *M. gallisepticum* positivity, which was verified by sequencing (Figure not shown).

### Treatment 

Tylosin tartrate 20% (w/w) (2.5 gm/l) (Rhotarsin 20 WSP, Rhone Ma, Malaysia) was utilized to treat CCRD in this patient, along with a combination of broad-spectrum bacteriostatic drugs streptomycin (25 mg/kg). Both antimicrobial drugs are regarded to be effective against *M. gallisepticum* and other opportunistic bacterial infections [7,8]. Additionally, amprolium (2 gm/5 l) was employed as a coccidiostat (Amprolox 20%, Ritma, Malaysia) [9]. For a week, all therapy was delivered via drinking water, and the death rate was dramatically improved upon re-visit by the veterinarian. Additionally, the farmer was urged to observe the withdrawal time before selling the birds in order to avoid antibiotic residues in the meat.

## Discussion

The farm was afflicted with CCRD and co-infected with coccidiosis, according to laboratory data. *M. gallisepticum* infection typically begins in young chicks and proceeds to the chronic stage, where secondary opportunistic bacterial infection occurs as a result of the compromised immune system. On this farm, the source(s) of *M. gallisepticum* infection was(were) not determined. However, the virus is considered to be transferred vertically (transovarially) from the breeder or during hatching [6,10]. While infected embryos may die, infected chicks may hatch and disseminate the virus throughout the flock [11]. Additionally, the infection could have been caused by inadequate maternal immunity transmitted from the mother to the chicks as a result of vaccination error or omission [12].

Second, *M. gallisepticum* can be spread horizontally by polluted water, feed, fomites, and the environment, as well as contact with wild birds [13,14]. However, *M. gallisepticum* infection can be latent, and clinical manifestations might vary significantly according to the incubation period, which can last from days to months. Overcrowding, suboptimal temperature, and a high ammonia concentration may all contribute to the bird’s vulnerability to illness [15]. Additionally, *M. gallisepticum* causes damage to the bird’s upper respiratory tract epithelial tissues, exposing it to secondary bacterial infections. Opportunistic infections such as pathogenic *E. coli* and other *Enterobacteriaceae* species from the gastrointestinal tract can infiltrate and infect many organs, producing strong endotoxins and causing septicemia, resulting in organ failure and death [16].

Along with CCRD, the birds were afflicted with mild coccidiosis. Although the actual *Eimeria* species was not determined in this case, it is extremely likely that *Eimeria acervulina* or *Eimeria maxima* was the causal agent, as evidenced by the presence of schizonts and macrogametes in the duodenal tissues [17]. Furthermore, both species were abundant in Malaysia, wreaking havoc on commercial broilers and rural hens [18,19].

In comparison to highly pathogenic species (*Eimeria tenella* and *Eimeria necatrix*), infection with *E. acervulina* or *E. maxima* is considered mild [20]. Coccidiosis can be contracted through polluted water and feed, as well as soiled floors with feces carrying oocysts, as a result of inefficient farm management and infection with other diseases [20].

Although CCRD and coccidiosis are considered common diseases in poultry [18,21], their co-occurrence in commercial broilers is uncommon in Malaysia. Antibiotics are typically used to control the CCRD, and the birds are sold as soon as they reach market age. However, the corpses’ value is frequently diminished as a result of lesions associated with secondary *E. coli* infections, as demonstrated in this example. Coccidiosis, on the other hand, is considered an opportunistic illness caused by moderately pathogenic *Eimeria* species (*E. maxima* or *E. acervulina*), which causes mild gastrointestinal lesions that farmers frequently neglect. Prolonged infection, on the other hand, can result in significant morbidity in birds, including impaired nutritional intake and growth retardation [22].

Several constraints were identified during the disease investigation. For example, we were unable to pinpoint the source of *M. gallisepticum* in this case, despite the fact that the source of infection can be multifactorial. Second, we were unable to identify the coccidia species in this case, which can be accomplished using a variety of molecular techniques, including the PCR assay and loop-mediated isothermal amplification [19]. This information would aid in early diagnosis, allowing for preventative measures to be done.

## Conclusions

CCRD and coccidiosis are frequent chicken diseases that can co-infect, resulting in increased morbidity and mortality in flocks. Early illness detection, sound farm management, and biosecurity all play a critical role in limiting disease transmission on the farm. Appropriate treatment should be begun promptly to alleviate the illness burden. Sick birds should be separated from the healthy flock and observed for a period of time following treatment. Additionally, seriously ill bids should be culled, and dead birds should be disposed of swiftly to prevent disease transmission. Finally, obtaining chicks from *M. gallisepticum*-free breeder flocks may help lower infection risk.

## List of Abbreviations

CCRD, Complex chronic respiratory disease; CRD, Chronic respiratory disease; PCR, Polymerase chain reaction.

## References

[1] Wakenell P (2016). Management and medicine of backyard poultry. Current Therapy in Avian Medicine and Surgery.

[2] Ramadan NM (2019). *Mycoplasma gallisepticum* overview in poultry. Am J Biomed Sci Res.

[3] Yasmin F, Ideris A, Omar AR, Hair-Bejo M, Tan SW, Tan CG (2014). Molecular detection of *Mycoplasma gallisepticum* by real-time PCR. J Vet Malay.

[4] Clark MI (2019). Management of breeding in small poultry production units. Veterinary Reproduction and Obstetrics.

[5] Bagal U, Dhaygude V, Kamd B, Mote C, Pawade M, Bhosale. (2019). Pathology and molecular diagnosis of *Mycoplasma gallisepticum* and *Mycoplasma synoviae *infections in broiler chickens from western Maharashtra, India. J Anim Res.

[6] Mettifogo E, Buzinhani M, Buim MR, Timenetsky J, Ferreira AJP (2015). Evaluation of a PCR multiplex for detection and differentiation of *Mycoplasma synoviae*, *M. gallisepticum*, and *M. gallisepticum* strain F-vaccine. Pesqui Vet Bras.

[7] Videnska P, Faldynova M, Juricova H, Babak V, Sisak F, Havlicova H (2013). Chicken faecal microbiota and disturbances induced by single or repeated therapy with tetracycline and streptomycin. BMC Vet Res.

[8] Farran MT, Ellakany HF, Shaib HA, Majed HM (2018). Evaluation of antibiotics to control *Mycoplasma gallisepticum* in broiler breeder chickens. Poult Fish Wildl Sci.

[9] Adewole SO (2012). The efficacy of drugs in the treatment of coccidiosis in chicken in selected poultries. Acad Res Int.

[10] Ricketts C, Pickler L, Maurer J, Ayyampalayam S, García M, Ferguson-Noel NM (2017). Identification of strain-specific sequences that distinguish a *Mycoplasma gallisepticum* vaccine strain from field isolates. J Clin Microbiol.

[11] Hruba H, Abdelsalam EE, Anisimov N (2020). Reproductive toxicity of fluoroquinolones in birds. J Avian Med Surg.

[12] Bradbury JM (2001). Avian mycoplasmas. Poultry diseases.

[13] Ley DH (2008). *Mycoplasma gallisepticum* infection. Disease of Poultry.

[14] Dhondt AA, DeCoste JC, Ley DH, Hochachka WM (2014). Diverse wild bird host range of *Mycoplasma gallisepticum* in eastern North America. PLoS One.

[15] Levisohn S, Kleven SH (2000). Avian mycoplasmosis (*Mycoplasma gallisepticum*). Rev Sci Tech.

[16] Stipkovits L, Kempf I (1996). Mycoplasmoses in poultry. Rev Sci Tech.

[17] Foreyt WJ (2001). Veterinary parasitology reference manual.

[18] Wan Norulhuda WAW, Nur Syakila MZ, Nik Kamarudin T, Norlida O, Saipul BR (2017). Coccidiosis in village chicken: a preliminary survey in Pasir Putih District, Kelantan, West Malaysia. Malays J Vet Res.

[19] Loo SS, Lim LS, Efendi NA, Blake DP, Kawazi SI, Wan KL (2019). Comparison of molecular methods for the detection of *Eimeria* in domestic chickens in Malaysia (Perbandingan Kaedah Molekul untuk Pengenalpastian *Eimeria* dalam Ayam Ternakan di Malaysia). Sains Malays.

[20] Adhikari A, Gupta R, Pant, GR. (2009). Prevalence and identification of coccidian parasite (*Eimeria *spp.) in layer chicken of Ratnanagar Municipality, Chitwan district, Nepal. J Nat Hist Mus.

[21] Tan CG, Jaganathan M, Ideris A, Abdul Rahman SO, Mutalib AR, Salim N (2016). Prevalence of *Mycoplasma gallisepticum *in commercial chickens and free flying birds. IOSR J Agric Vet Sci.

[22] Willis GM, Baker DH (1981). *Eimeria acervulina* infection in the chicken: a model system for estimating nutrient requirements during coccidiosis. Poult Sci.

